# Novel insight into metabolic reprogrammming in cancer radioresistance: A promising therapeutic target in radiotherapy

**DOI:** 10.7150/ijbs.79928

**Published:** 2023-01-01

**Authors:** Yilin Yu, Jie Yu, Shengfang Ge, Yun Su, Xianqun Fan

**Affiliations:** Department of Ophthalmology, Shanghai Key Laboratory of Orbital Diseases and Ocular Oncology, Shanghai Ninth People's Hospital, Shanghai JiaoTong University School of Medicine, Shanghai, P.R. China.

**Keywords:** Cancer, Radioresistance, Metabolic Reprogrammming, Therapeutic Targets

## Abstract

Currently, cancer treatment mainly consists of surgery, radiotherapy, chemotherapy, immunotherapy, and molecular targeted therapy, of which radiotherapy is one of the major pillars. However, the occurrence of radioresistance largely limits its therapeutic effect. Metabolic reprogramming is an important hallmark in cancer progression and treatment resistance. In radiotherapy, DNA breakage is the major mechanism of cell damage, and in turn, cancer cells are prone to increase the metabolic flux of glucose, glutamine, serine, arginine, fatty acids etc., thus providing sufficient substrates and energy for DNA damage repair. Therefore, studying the linkage between metabolic reprogramming and cancer radioresistance may provide new ideas for improving the efficacy of tumor therapy. This review mainly focuses on the role of metabolic alterations, including glucose, amino acid, lipid, nucleotide and other ion metabolism, in radioresistance, and proposes possible therapeutic targets to improve the efficacy of cancer radiotherapy.

## Background

Cancer is currently a leading cause of death worldwide, killing millions of people every year 1. As one of the most important treatments for cancer, radiotherapy kills cancer cells mainly through reactive oxygen species (ROS) production and DNA damage 2. However, tumor cells can develop radioresistance through multiple mechanisms including DNA damage repair, antioxidation, cell cycle arrest, accumulation of cancer stem cells (CSCs), and immunosuppressive microenvironment formation 2-4, leading to cancer recurrence and metastasis after radiotherapy. The resistance of cancer cells to radiotherapy is a huge obstacle to the effective control of cancer development.

Metabolic reprogramming, the adaptive alteration of metabolic pathways in tumor cells, is considered one of the hallmarks of malignancy 5. The exploration of tumor metabolism dates back to the “Warburg effect” proposed by German biochemist Dr. Warburg in the 1920s 6, which indicated the importance of glycolysis in cancer cells and pioneered the study of tumor metabolism 6, 7. Afterward, scientists gradually discovered other metabolic reprogramming pathways in cancer cells, such as nucleotide metabolism 5. More than 70 years ago, American pediatric pathologist Sidney Farber discovered that the folic acid antagonist aminopterin could induce complete remission in children with acute lymphoblastic leukemia (ALL) 8. This landmark research provided a foundation for cancer chemotherapy based on inhibiting the biosynthesis of nucleotides 8, 9. Today, many antimetabolites, such as 5-fluorouracil/pemetrexed and methotrexate, have been approved and used in cancer treatment 9. In particular, the approval of ivosidenib and enasidenib, which target to mutant isocitrate dehydrogenase (IDH) in acute myeloid leukemia (AML), has become another milestone in precision tumor therapy that targets aberrant metabolism 10. Metabolic reprogramming can be induced by aberrant activation of oncogenes, signaling pathways and epigenetic modifications, influencing the progression of cancer cells 5, 11, 12. Previous studies have discovered that MYC, AKT, ERK are associated with enhanced glycolysis and nucleotide synthesis in cancer cells 5, 11, 13, 14. Histone lactylation, elevated in tumors, is associated with poor prognosis of ocular melanoma as well 15. Apart from tumor cells, noncancer cells in the TME including endothelial cells, fibroblasts, and immune cells are also involved in cancer development through metabolic reprogramming 16-18. For instance, activation of p38α-MAPK in cancer-associated fibroblasts mobilizes glycogen in tumor cells and promotes its metastasis 18. Furthermore, metabolic reprogramming is also linked to chemo- and immune-therapy resistance. Van Gastel N et al. found that after chemotherapy, AML cells developed chemoresistance by activating glutamine metabolism to promote pyrimidine synthesis 19. Effector Treg cells take up lactate through MCT1 to enhance PD-1 expression and remodel its function and phenotype, which may be one of the reasons for resistance to PD-1 immunotherapy 16.

In radiotherapy DNA breakage is the main mechanism of radiation-induced cell damage, in turn, cancer cells upregulate the metabolic flux of glucose, amino acids, fatty acids etc., providing sufficient substrates and energy for DNA damage repair. Similarly, in response to radiation-induced ROS, tumor cells develop a set of antioxidant strategies. For example, radiation-induced hypoxia stimulates serine metabolism through HIF-1/2 to increase NAPDH/GSH levels, enhancing tumor antioxidant capacity 20. By integrating of machine learning and experimental validation, Joshua E. Lewis et al. demonstrated that many of the metabolites involving glucose metabolites (e.g., fructose 1,6-bisphosphate, 3-phosphoglyceric acid, succinyl-CoA, succinate), lipids and fatty acid metabolites were all positively correlated with radioresistance 21, suggesting that metabolic reprogramming plays a vital role in radiation resistance. Therefore, targeting tumor metabolism may provide a promising therapeutic strategy for radiosensitization. In this review, we elaborate on the role of several basic metabolic alterations (e g. glucose metabolism, amino acid metabolism, lipid metabolism and nucleotide metabolism) in tumor radioresistance, and propose therapeutic prospects for targeting metabolic reprogramming in radiotherapy.

## Glucose metabolism and cancer radioresistance

**Figure 1:**
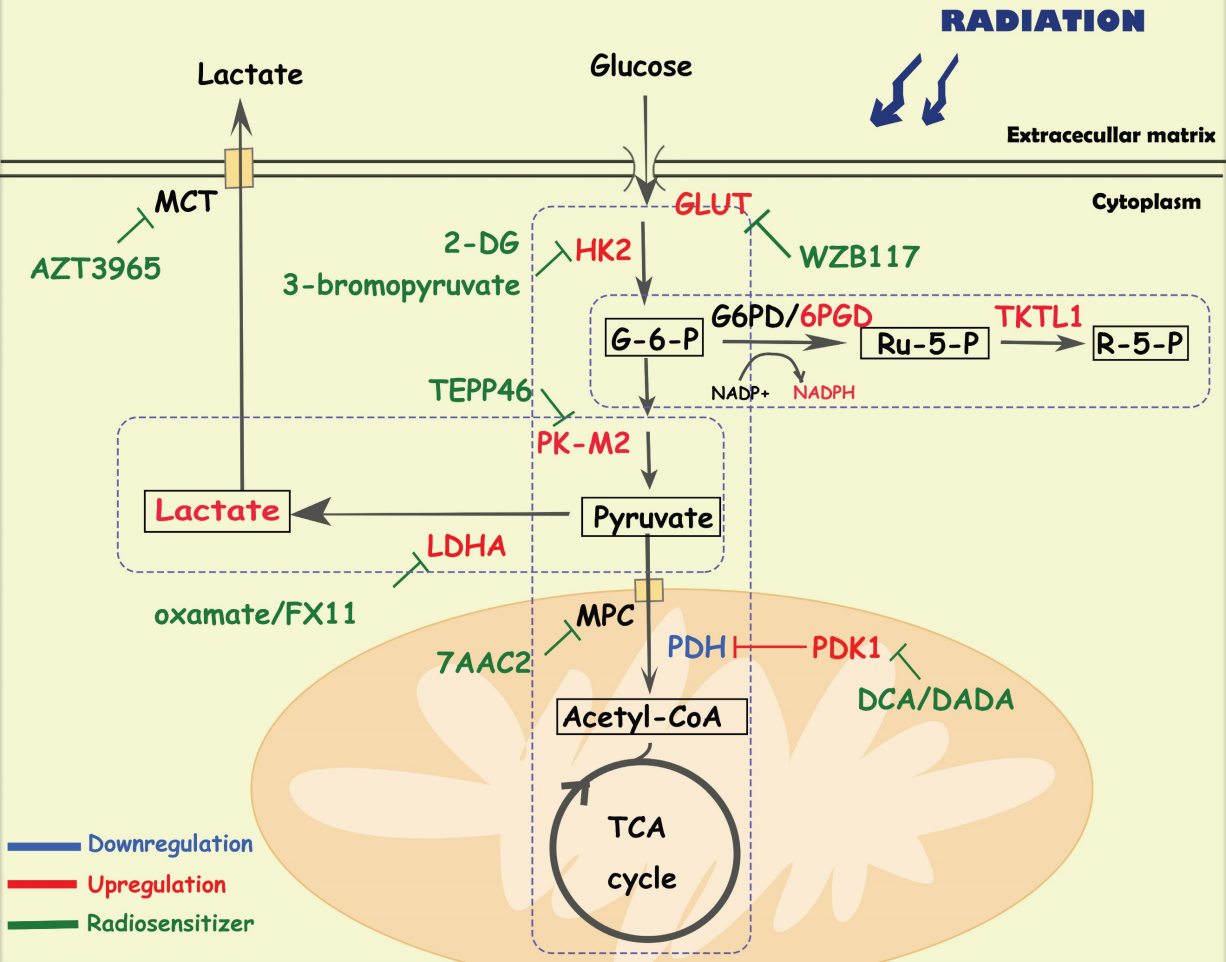
** Diagram of glucose-related metabolic pathways involved in cancer radioresistance and its radiosensitizers.** In response to radiation, cancer cells increase glucose flux via upregulating GLUT and HK2, facilitating the glucose metabolism including glycolysis, OXPHOS and PPP. In activated aerobic glycolysis, PK-M2/ LDHA/lactate are the major up-regulated metabolites and metabolic enzymes. In OXPHOS, metabolic enzymes (e g., PDH/MPC) and the integrity of mitochondrial function also play an important role in cancer radioresistance. In PPP, overexpression of 6PGD/ TKTL1 enhance the production of NAPDH and nucleotides.

Glucose provides energy and a carbon skeleton for biosynthesis. Cancer cells have elevated glucose uptake, which can be detected by FDG-PET (fluorodeoxyglucose-positron tomography) and used for cancer identification and treatment assessment 22, 23. Active aerobic glycolysis is one of the hallmarks of metabolic reprogramming in several cancers, contributing to treatment resistance and recurrence 5, 24, 25. In addition to glycolysis, the pentose phosphate pathway (PPP), and oxidative phosphorylation pathways (OXPHOS) are also reprogrammed to protect tumor cells from radiation (**Figure 1**).

### Aerobic glycolysis

Aerobic glycolysis is a crucial alteration in cancer glucose metabolism reprogramming, and is characterized by an increased glucose uptake rate and high lactic acid production. Tumor cells take up glucose through glucose transporters (GLUTs) and convert it to glucose-6-phosphate(G-6-P) via hexokinase 2 (HK2). Then, G-6-P is converted to pyruvate through a series of enzymatic reactions, in which the M2 isoform of pyruvate kinase (PKM2) plays a key role. Finally, lactic acid is generated by lactate dehydrogenase (LDHA). Lactate and pyruvate are transported by monocarboxylate transporters (MCTs) and mitochondrial pyruvate carriers (MPCs) in the cell membrane and mitochondrial membrane. Glycolysis plays an important role in tumor initiation and progression. For instance, Liu Y et al. found that tumor cells regulate glycolytic metabolism through FTO-mediated m6A modification to promote the activation and function of T cells, protecting tumor cells from immune surveillance 26. Recently, scientists have been seeking the precise treatment of cancer by inhibiting the activity of key enzymes in the glycolysis pathway 9.

Recent studies have indicated a positive correlation between glycolysis and radioresistance in multiple malignant tumors including pancreatic cancer, prostate cancer and laryngeal carcinoma 23, 25, 27. GLUT1 overexpression is associated with radioresistance and poor prognosis in patients with oral and head and neck squamous cell carcinomas 28. Upregulation of GULT1 expression was also observed in radioresistant tumor cells, which was related to oncogene activation, hypoxia stimulation, and regulation of signaling pathways such as PI3K/AKT 25. Radiotherapy combined with GLUT1 inhibition sensitized tumor cells 29-31. For example, in subcutaneous glioma cells treated with radiotherapy, the GLUT1 inhibitor apigenin reduced cell stemness and DNA damage repair by inhibiting the NF-κB/hif-1α/GLUT1 pathway, facilitating radiosensitivity 30. WZB117, a specific inhibitor of GLUT1 sensitized radioresistant breast cancer cells to irradiation 31. Moreover, in cervical cancer cells, treatment with HK2 inhibitor 2-DG delayed the proliferation of radioresistant cells and sensitized them both in vitro and in vivo 32. 3-bromopyruvate, a HK2 inhibitor, improved the sensitivity of pancreatic cancer cells to irradiation by reduce nucleotide biosynthesis and enhance DNA damage 33.

PKM2, the main isoform expressed in tumor cells 34, is responsible for converting phosphoenolpyruvate into pyruvate. Wu S et al. found that the phosphorylation level of PKM2 s222 is related to the radioresistance and prognosis of glioblastoma 35. Mechanistically, stimulated by radiation-induced DNA damage, PKM2 is phosphorylated at serine(s)222 and interacts with the FACT complex and histone chaperone, facilitating γH2AX-mediated chromatin loading, which promotes DNA damage repair and cell survival of tumor cells 35. Silencing the expression of PKM2 inhibited the phosphorylation of AKT and PDK1, sensitizing non-small cell lung cancer cell lines and xenografts to radiotherapy by enhancing apoptosis and autophagy 36. Combining PK-M2 activators (TEPP486) with radiation to enhance the effect of radiotherapy in resistant cancers, such as TNBC 37.

Elevated LDHA expression is also associated with poor prognosis of patients with multiple cancers (e g. head and neck squamous cell carcinoma, prostate cancer, non-small cell lung cancer) and has a positive correlation with radioresistance 38-41. Indeed, LDHA5 overexpression suggests a hypoxic state in cancer cells, which is associated with cancer recurrence, metastasis and radioresistance 38, 39. Treatment with LDHA inhibitors (e g, oxamate 41) can overcome radioresistance by enhancing radiation-induced DNA damage and apoptosis. The long noncoding RNA LINC00518, an oncogene in cutaneous malignant melanoma, also induced radioresistance via a miR-33a-3p/ HIF-1α/LDHA negative feedback loop 42.

Lactic acid acts not only as a metabolic fuel for tumor tissues, but also a signaling molecule associated with the immunosuppressive microenvironment, which contributes to cancer proliferation, invasion, metastasis and radioresistance 43-45. Rather than affecting tumor cells, lactate tends to influence the metabolic phenotype of nontumor cells in the TME, mediating immune escape 46. Yang X et al. found that radiation-upregulated lactate enhanced the immunosuppressive activity of myeloid-derived suppressor cells (MDSCs), which inhibited antitumor T-cell activity and accumulation in the TME, accelerating the progression of pancreatic cancer 46. Blocking lactate secretion or knocking out Hif-1α in MDSCs could reverse these responses and effectively inhibit cell progression after radiotherapy 46. In addition, MCT and MPC, the major transporters of lactate, also play important roles in radioresistance. The combination of the MCT1 inhibitor AZD3965 and radiotherapy showed a greater therapeutic effect in small cell lung cancer xenografts than radiotherapy alone, suggesting that MCTs may be a potential target for radiosensitization 47. Interfering with the expression of MPC1 also contributed to cancer epithelial-mesenchymal transition and radioresistance 48. In cervical cancer xenograft models, 7AAC2, a novel MPC1 inhibitor, combined with radiation therapy significantly decreased tumor growth compared with 7AAC2 or radiation therapy alone 49.

### Oxidative phosphorylation

Under physiological conditions, pyruvate in mitochondria is converted into acetyl-CoA and then enters the tricarboxylic acid cycle to produce ATP through OXPHOS, which satisfies more than 95% of cell energy demands. Indeed, tumor cells can adapt to altered metabolic environments by switching between glycolysis and OXPHOS. Increased OXPHOS and NADH/NAD+ metabolism mediated by mitochondrial membrane fusion contributes to tumor immortalization 50. Age-related mitochondrial OXPHOS deficiency promotes intestinal cancer cell growth and survival by upregulating the serine de novo synthesis pathway (SSP) 51. Inhibition of OXPHOS with gboxin restricts glioblastoma growth by inhibiting the activity of ATP synthase 52.

Pyruvate dehydrogenase (PDH) is responsible for the conversion of pyruvate to acetyl-CoA. Pyruvate dehydrogenase kinases (PDKs) inhibit the activity of PDH, which is a key enzyme regulating the switch between oxidative phosphorylation and glycolysis in cancer cells. Previous studies have indicated that PDK1 and phospho-pyruvate dehydrogenase (p-PDH) are positively associated with radioresistance 53-55. In complex I-deficient mitochondrial cells, there was an increase in the expression of p-PDH, accompanied by radioresistance by reducing histone acetylation and promoting DNA damage repair 53. Dichloroacetate (DCA), a specific inhibitor of PDK, can not only downregulate p-PDH levels, extracellular acidification rates and lactate production, but also significantly increase ROS levels in triple-negative breast cancer cells, leading to radiosensitization 54. Moreover, diisopropylamine dichloroacetate (DADA), an inhibitor of PDK, enhanced radiosensitization in esophageal squamous cell carcinoma by increasing mitochondria-derived ROS levels 56.

### Pentose phosphate pathway

In the pentose phosphate pathway (PPP), nicotinamide adenine dinucleotide phosphate (NADPH) and ribose 5-phosphate (R-5-P) are the main metabolites, the former can protect cells from ROS damage, and the latter is the essential material for nucleotide synthesis. The PPP can be divided into two parts: the oxidative portion is controlled by the key enzymes glucose-6-phosphate dehydrogenase (G6PD) and 6PGD, which are responsible for the production of NAPDH; and the non-redox portion is controlled by transketolase-like protein 1 (TKTL1), which is mainly responsible for the production of ribose. Studies have demonstrated upregulation of the PPP in several cancer types. Lin R et al. found that suppression of 6PGD decreased lipogenesis, and RNA biosynthesis and elevated ROS levels in cancer cells, which attenuated cell proliferation and tumor growth, suggesting that 6PGD could be an anticancer target 57. Increased flux of the PPP was also confirmed in radioresistant head and neck squamous cell carcinoma cells 58. The EGFR signaling pathway mediates the phosphorylation and activation of 6PGD, which promotes the metabolic flux of the PPP, and DNA synthesis and repair, facilitating tumor growth and radioresistance 59. Moreover, suppression of TKTL1 increased the levels of ROS and sensitized tumor cells to ionizing radiation, indicating that TKTL2 may be a potential therapeutic target 60.

## Amino acid metabolism and cancer radioresistance

Essential amino acids (histidine, lysine, methionine, phenylalanine, threonine, tryptophan, and branched-chain amino acids) cannot be produced by the de novo pathway in the human body. Cancer cells capture amino acids via transporters for biomacromolecules and energy production. Amino acid metabolism reprogramming significantly affects tumor cells and their immune microenvironment. In a leukemia stem cell (LSC) population, Jones CL et al. demonstrated the upregulation of the nutrient uptake rate, steady-state levels, and catabolism 61. LSCs isolated from AML patients are uniquely reliant on amino acid metabolism for survival, which can be reversed by pharmacological inhibition of amino acid metabolism 61. In addition, tumor cells are dependent on an exogenous supply of amino acids, suggesting that limiting the availability of these nutrients can selectively kill tumor cells.

### Glutamine metabolism

Glutamine (Gln) is the most essential nutrient for intracellular energy production, redox homeostasis and biosynthesis in cells 62. Gln is transported into cells by transporters (e.g. ASCT2/SLC1A5/SLC7A5/LAT1) and converted to glutamate (Glu) by mitochondrial glutaminase (GLS1/GLS2) 63. Glu in mitochondria is then converted to α-ketoglutarate, an intermediate product of TCA, by glutamate dehydrogenase (GDH) or transaminase (TA). Additionally, in the cytoplasm, Glu can also be used to synthesize nucleotides, simultaneously producing glutathione (GSH) to maintain redox homeostasis and avoid oxidative stress 62. Cells can also produce Gln from NH_4_ and Glu via the catalysis of glutamine synthetase (GS), participating in the transport of ammonium ions 63, 64. Active glutamine metabolism provides sufficient carbon and nitrogen sources to promote tumor growth and proliferation 65. Rather than glucose, cell-intrinsic programs drive the preferential acquisition of glutamine by cancer cells 66. Additionally, multiple cancer cells (including breast cancer, pancreatic CSCs and GBM) received radioresistance in response to glutamine deprivation, suggesting a significant combination between glutamine metabolism and radioresistance 64, 67-70.

It has been reported that radiation increases the activity of GLS in multiple cancer cells such as head and neck squamous cell carcinoma, lung cancer, prostate cancer and cervical cancers, contributing to radioresistance 71-74. In radioresistant cervical cancer tissues, GLS2 was overexpressed, and silencing GLS2 could reverse this radioresistant phenotype by decreasing GSH and NADH levels 73. High requirements for glutamine are found in radioresistant prostate cancer (PCa) cells and CSCs 74. Overexpression of GLS and MYC improved GSH production and maintained the redox state, which was significantly associated with a reduction in progression-free survival in PCa patients who received radiotherapy 74. Glutamine catabolism regulates the epigenetic reset of chromatin-modifying dioxygenases by α-KG as well, contributing to the maintenance of CSCs 74. Interestingly, activation of ATG5-mediated autophagy in the absence of glutamine is another tumor survival strategy to defend against radiation-mediated cellular damage 74. In addition, Binkley MS et al. reported that GLS inhibition preferentially radiosensitizes KEAP1-mutant non-small cell lung cancer cells via depletion of GSH and enhanced DNA damage 75. GLS inhibition also specifically sensitized IDH-mutant glioma cells to oxidative stress and radiation in vitro and in vivo 76. Telaglenastat (CB-839), a specific GLS inhibitor, induced radiosensitization by enhancing oxidative stress in cervical cancer and head, neck squamous cell carcinoma and breast cancer 70, 71, 77. Currently, the GLS1 inhibitor CB-839 is under phase 1 clinical evaluation in patients with advanced solid tumors (e.g., NCT02071862, NCT03875313, and NCT02861300), showing encouraging treatment effects, and further clinical trials are needed to establish its utility as a radiosensitizer 70, 76.

Several studies have indicated that high expression of GS contributes to radioresistance of cancer cells, such as lung cancer, pancreatic cancer and glioblastoma cells 64, 77-79. High GS activity is associated with radioresistance in cancer cells through the regulation of nucleotide metabolism, G2/M recovery and DNA repair 79. Interestingly, a recent study suggested that in addition to participating in glutamine metabolism, GS can directly interact with the nuclear pore protein NUP88, activating the anaphase-promoting complex (APC/C^CDC20^) to ensure the process of cell division and promote the proliferation of cancer cells, indicating a new function of GS 80. It is worth mentioning that cancer cells can simultaneously overexpress GS and GLS sometimes, which may create an ineffective cycle. Therefore, how tumor cells dynamically regulate the expression of GS and GLS to maintain the growth of malignant tumor cells is an important issue in tumor metabolism.

### Serine/glycine metabolism

Serine/glycine metabolism provides one-carbon units for the synthesis of proteins, lipids, and nucleic acids, which are essential for tumor growth and homeostasis. Cancer cells take up serine/glycine mainly from the extracellular microenvironment, as well as from de novo synthesis. In serine/glycine metabolism, tumor cells synthesize and utilize serine/glycine by a phosphoglycerate dehydrogenase (PHGDH)/phosphoserine aminotransferase 1 (PSAT1)/phosphoserine phosphatase (PSPH)/hydroxymethyltransferase (SHMT)-mediated continuous enzymatic reaction. To our knowledge, serine dependent-single carbon metabolism is closely related to nucleotide synthesis and is crucial to the proliferation of tumor cells 81. Serine metabolism supports macrophage produce IL-1β, which contributes to the immunosuppression in breast cancer 82, 83. Suppressing serine metabolism enhances the polarization of interferon-γ-activated macrophages (M(IFN-γ)) but suppresses that of interleukin-4-activated macrophages (M(IL-4)) by regulating the IGF1-p38 axis 84. Recent evidence suggests that serine/glycine metabolism is not only an important driver of macrophage function 82-84 and revascularization 85, 86, but also contributes to the maintenance of CSCs in the TME. Silencing of PHGDH leads to downregulation of transcriptional regulators involved in self-renewal and pluripotency (e g. Oct4, Nanog, Sox2, Bmi1), reducing the formation of embryonic and breast CSCs 87.

Activation of serine/glycine metabolism could be a strategy for radioresistant cells to survive and recover during radiotherapy. After radiotherapy, the metabolome of B16 melanoma cells and the breast cancer cell line HCC1937 showed an increased level of glycine 88, 89. Similarly, increased circulating levels of serine, leucine, and isoleucine in serum were observed in breast cancer patients who received local radiotherapy after tumor resection 90. In resistant head and neck squamous cell carcinoma, activation of de novo serine/glycine biosynthesis has been observed 91. Previous studies found that serine starvation combined with metformin and benzformin inhibited mitochondrial OXPHOS by targeting ETC complex I, facilitating radioresistance^92^-^94^.

Hypoxia is one of the predisposing factors of radioresistance. It has been reported that under hypoxic conditions, the HIF-1/HIF-2 and PERK-eIF2α-ATF4 signaling pathways are activated, which induces upregulation of SHMT2/PHGDH/PSAT1/PSPH levels, increasing de novo serine/glycine biosynthesis 20, 95-97. As glycine is one of the substrates for GSH synthesis, increased serine/glycine metabolism resulted in the accumulation of NAPDH/GSH, promoting the elimination of ROS. Serine/glycine metabolism is also associated with DNA stability. In neuroendocrine prostate tumors, loss of tumor suppressor protein kinase C (PKC) λ/ι enhanced serine metabolism through mTORC1/ATF4/PHGDH axis, which leads to aberrant DNA methylation by increased one-carbon metabolic flux, ultimately leading to treatment resistance and poor prognosis 98. Wilson JL et al. found that PHGDH, as a metabolic checkpoint of M2 macrophages, induced the polarization of macrophages from M1 to M2, which are associated with radioresistance and relapse 99-101.

### Arginine metabolism

Arginine is synthesized from citrulline by argininosuccinate synthase 1 (ASS1) and argininosuccinatelyase (ASL), and converted to ornithine and urea by arginase (ARG). ASS1 deficiency is common in many cancers, reported as arginine auxotrophic tumors, in which cancer cells are unable to synthesize endogenous arginine, resulting in an increased dependence on extracellular arginine 102, 103. Arginine deiminase (ADI) and ARG are responsible for depleting extracellular arginine, resulting in antitumor activity. Therefore, enzymotherapeutic arginine deprivation therapy (ADT) has become a new metabolic strategy for cancers, and has already been proven to effectively inhibit the growth of cancers in both preclinical studies and clinical phase I-III trials 103-105.

Arginine metabolism is also associated with radiotherapy efficacy. In mice, overexpression of ARG1 in myeloid cells limits the control of residual disease after radiotherapy of tumors 106. MDSCs have been reported to deplete L-arginine in the TME by overexpressing ARG1, which promotes the apoptosis of antitumor T cells and macrophages 107. According to the metabolic characteristics of arginine in cancer cells, researchers invented D-arginine-loaded metal-organic framework nanoparticles, which can be used to sensitize osteosarcoma to radiotherapy 108. Previous studies have shown that ADT can enhance radiosensitivity in certain tumors, even in ASS-positive colorectal cancer cells 109-111. For example, in glioblastoma cells, ADT combined with radiotherapy improved the survival of tumor cells, especially in TP53-mutated and-knockdown cells 103. Vynnytska-Myronovska B et al. found that arginine withdrawal alone or in combination with the arginine natural analog canavanine is a promising antitumor strategy with the potential to enhance cancer cure by radiotherapy 110. Recently, another opposing strategy for radiosensitization in solid tumors was reported. Rossella Marullo et al. demonstrated that administration of oral L-arginine induced radiosensitization and local control in patients who underwent standard radiation therapy 112, which is associated with increased DNA damage via nitric oxide (NO)-mediated metabolic suppression 112. It is worth mentioning that NO plays a dual role in tumor cells. An appropriate amount of NO can promote tumor growth; however, either too low or too high a level of NO will limit the proliferation of tumor cells. Since arginine is a precursor to NO 112, 113, it may explain the contradiction between ADT and oral L-arginine.

### Other amino acid metabolism

#### Tryptophan metabolism

Tryptophan (Trp) is an essential amino acid ingested from the diet, and 95% of free Trp is catabolized through the kynurenine pathway and is involved in the regulation of immunity, neuronal function, and intestinal homeostasis. The imbalances of Trp metabolism in cancer have drawn great interest in targeting the kynurenine pathway for therapy, especially the rate-limiting enzymes indoleamine-2,3-dioxygenase 1 (IDO1), IDO2, tryptophan-2,3-dioxygenase (TDO) and kynurenine monooxygenase (KMO) 114, 115. It has been found that IL4I1, a metabolic enzyme produced by tumors, breaks down tryptophan to activate aryl hydrocarbon receptors, enhancing tumor aggressiveness and suppressing antitumor immunity 116. In addition, it has been reported that in UM-SCC-74A cells, a radiosensitive uveal melanoma cell line, the most pronounced metabolic change after irradiation is related to tryptophan metabolism 91. IDO1 inhibitors, Epacadostat and INCB023843, are proved to be potential radiosensitivity agents 117, 118. Recently, researchers have developed a nanomaterial delivery tool “coordination polymer-coated CaCO3” targeting IDO, which can reinforce radiotherapy by reprogramming the immunosuppressive metabolic microenvironment 119.

#### Asparagine metabolism

Mammalian cells synthesize asparagine from aspartate and glutamine by asparagine synthase (ASNS). Currently, the first antitumor drug that directly targets aspartate metabolism is L-asparaginase (L-ASPase) from *Escherichia coli* and *Erwinia europaea*, which has been approved by the FDA for the clinical treatment of acute lymphoblastic leukemia 120. Asparagine can increase the synthesis of purine and pyrimidine by activating mTORC1 signaling when mitochondrial respiration is impaired, and promote tumor cell proliferation 121. Elevated ASNS expression brought about antioxidative stress and rapid transformation between glycolysis and oxidative phosphorylation, leading to enhanced proliferation and radioresistance in tumor cells 122.

## Lipid metabolism and cancer radioresistance

Lipids are not only important building blocks of biological membranes, but are also used for energy storage, biosynthesis and signaling pathways. Dysregulation of lipid metabolism, especially fatty acid (FA) metabolism, is an important metabolic phenotype alteration in cancer cells 123 and exhibits flexibility and vulnerability 124. Lipid metabolism reprogramming influences cancer cell growth and migration, neovascularization, evasion of immune surveillance, and treatment resistance. For instance, Lim SA et al. demonstrated that inhibiting lipid synthesis in Treg cells unleashes effective antitumor immune responses without autoimmune toxicity, pointing to new mechanism of targeting lipid metabolism 125.

### Fatty acid biosynthesis

Fatty acid synthesis in normal tissues is mainly restricted to hepatocytes and adipocytes. However, even in the presence of an exogenous lipid source, cancer cells activate lipogenesis in response to high metabolic demands, which is one of the characteristics of cancer cells and normal cells 124, 126. The fatty acid metabolism of tumor cells involves glucose metabolism as well. The main substrate for FA synthesis is acetyl-CoA, which is synthesized from extracellular nonlipid substrates (e g. acetate) or citrate by ATP-citrate lyase (ACLY) and acetyl-CoA synthetase 2 (ACSS2). Afterward, acetyl-CoA is converted to FAs through a series of enzymatic reactions, in which acetyl-CoA carboxylase (ACC), fatty acid synthase (FASN) and stearoyl-CoA desaturase (SCD) are the main rate-limiting enzymes 127. Previous studies have reported that overexpression of ACLY/ACSS2/ACC/FASN/SCD promotes tumor cell proliferation, invasion and metastasis in various types of cancer 128-131. Moreover, in liver cancer cells and lung cancer cells with SCD suppression, cancer cells can secrete an uncommon unsaturated fatty acid, sapienate, through FADS2, which is mostly secreted by sebaceous glands in normal tissues 132.

In radioresistant tumor cells, FA synthesis is increased as well, producing more plasma membrane phospholipids and signaling molecules 128. Göttgens EL et al. revealed that high expression of ACLY was associated with poor overall survival in HNSCC patients who received radiotherapy 127, and inhibition ACLY expression caused an impairment of DNA damage repair, leading to radiosensitization in HNSCC cell lines 127. Moreover, elevated expression and activity of FASN have been observed in radioresistant nasopharyngeal and pancreatic carcinoma cells, and were positively correlated with poor prognosis of patients 133, 134. In prostate cancer cells, overexpression of FASN upregulated androgen receptors via the Akt/NF-κB pathway, leading to cell cycle arrest and DNA repair enhancement, ultimately inducing radioresistance 135, 136. Upregulation of FASN also induced radioresistance in nasopharyngeal carcinoma cells by frizzled class receptor 10^137^. Additionally, FASN inhibitors, such as EGCG (epigallocatechin gallate) and orlistat, were used as radiosensitizers to improve the efficacy of radiotherapy 135-137.

### Fatty acid oxidation

In addition to providing essential phospholipids and signaling molecules for tumor growth, FAs also enter the mitochondria for β-oxidation, known as fatty acid oxidation (FAO). First, FAs are converted to acyl-CoA by the acyl-CoA synthase family (ACS). Then acyl-CoA is transported into mitochondria through carnitine palmitoyltransferase (CPT) on the mitochondrial membrane. Under the catalysis of the FAO system, acyl-CoA undergoes four consecutive reactions with dehydrogenation, water addition, re-dehydrogenation and thiolysis, and finally generates acetyl-CoA 138, 139. Increased FAO activity has been observed in multiple cancers, such as lung cancer, triple negative breast cancer and glioma, to meet energy demands 140. Enhanced FAO metabolism is required for the differentiation and activation of tumor-associated macrophages 141. Duman C et al. demonstrated that acyl-CoA-binding protein drives glioblastoma tumorigenesis by sustaining fatty acid oxidation 139. The nuclear receptor Nur77, which is essential for melanoma cells to adapt to the metabolic stress of glucose starvation, promotes FAO by protecting TPβ from oxidative inactivation, thereby maintaining ROS levels in cells to facilitate cell survival 142.

Active FAO metabolism and overexpression of FAO-related enzymes are also observed in radioresistant tumor cells 123. In radioresistant breast cancer cells, CD47 expression is regulated by FAO metabolites, enabling tumor cells to escape phagocytosis by macrophages 143. The combination of FAO inhibitors ((e.g., the CPT1 inhibitor etomoxir) and anti-CD47 antibodies improved tumor control in a mouse model of GBM that relapsed after radiotherapy 143. Furthermore, during radiotherapy, lipids released by dead tumor cells can provide a survival advantage for CSCs, promoting the accumulation of CSCs in radioresistant tumors through FAO-mediated self-renewal and metastasis 144. Long-chain acyl-CoA synthase (ACSL), a member of the ACS family, has been reported to mediate radioresistance by regulating FOXM1 to enhance DNA damage repair and inhibit apoptosis 145. Tan Z et al. found that FAO was active in radiation-resistant nasopharyngeal carcinoma cells, and its rate-limiting enzyme CPT1A expression was also upregulated in these cells, which was significantly associated with overall survival after radiotherapy in nasopharyngeal carcinoma patients 146. Mechanistically, CPT1A promotes fatty acid transportation by interacting with rab14, reducing radiation-induced lipid accumulation, and inducing radioresistance 146. Another study also reported that the PGC1α/CEBPB/CPT1A axis promotes radiation resistance in nasopharyngeal carcinoma by activating FAO 147. In radioresistant breast cancer cells and radioresistant breast CSCs, blocking FAO via an FAO inhibitor or by CRISPR-mediated CPT1A/CPT2 gene deficiency inhibited radiation-induced ERK activation and aggressive growth and radioresistance 148.

### Arachidonic acid metabolism

Tumor cells utilize phospholipids to synthesize arachidonic acid (AA) through cytosolic phospholipase A2 (cPLA2), and then AA is converted to biologically active substances (e g. leukotrienes, prostacyclins, prostaglandins, thromboxanes) through lipoxygenases (LOXs) and cyclooxygenases (COXs), which contribute to the human cardiovascular system and immune system. In recent years, it has been found that arachidonic acid metabolic pathways also play an important role in tumors 149. For instance, Liao P et al. found that CD8+ T-cell derived interferon (IFNγ) combined with AA induced tumor ferroptosis 150. PIK3CA mutations drive multiple signaling networks involving mTORC2-PKCζ-mediated activation of cPLA2, and cPLA2 inhibition and dietary fat restriction restore tumor immune cell infiltration and inhibit breast tumor growth 149. In addition, aspirin, a nonsteroidal anti-inflammatory drug targeting COXs, has been shown to anti-tumor effect 151, 152. Professor John Burn's team at Newcastle University, UK, found that in high-risk groups of bowel cancer patients carrying hereditary mutations, taking aspirin for more than 2 years could reduce the incidence of bowel cancer by 50%, and this protective effect can last for up to 20 years 153.

By analysis of metabolic biomarkers from the multiomics classifier for radiation response, Joshua E. Lewis et al. found increased production of membrane phospholipids and arachidonic acid precursors, resulting in significant correlations between inflammation-mediating eicosanoids and radioresistance 21. In GBM, prostaglandin E2 (PGE2), a major metabolite produced from AA metabolism, upregulated Id1 through the MAPK signaling pathway, inducing radioresistance 154. Overexpression of cPLA2, LOXs and COXs also contributes to the radioresistance of tumors 154-156. It has been reported that overexpression of 15-LOX-1 in colorectal cancer cells exposed to radiation induces radiation resistance through downregulation of MacroH2A2 157. Additionally, 12-LOX inhibitors (baicalein or bmd122) have been demonstrated to sensitize prostate cancer cells to radiation 158. COX-2 inhibitors have shown radiosensitization effects in multiple cancers including renal cell carcinoma, breast cancer, cervical cancer, and non-small cell lung cancer 159-162. A phase I clinical trial of patients with recurrent head and neck cancer demonstrated that the combination of COX-2 inhibitors (erlotinib, celecoxib) and radiotherapy can improve progression-free survival (37%) and overall survival (55%) 163.

### Cholesterol biosynthesis

Cholesterol, an important component of the membrane structure, is also used as a precursor to synthesize other substances such as bile acids, steroid hormones, vitamins, and oxidized cholesterol. Cholesterol metabolism in tumor cells is characterized by upregulation of cholesterol synthesis and uptake, and enrichment of various cholesterol derivatives 162. Microarray analysis revealed that many genes involved in cholesterol synthesis (e.g., FDPS, ACAT2, AG2, SLC12A2) were consistently associated with radioresistance in cell lines 164. In cholesterol biosynthesis pathways, 3-hydroxy-3-methylglutaryl-CoA reductase (HMGCR) is the rate-limiting enzyme in in the conversion of HMG-CoA to mevalonate, whose expression is upregulated in gastric cancer, glioblastoma and prostate cancer. Pitavastatin (Livalo), an HMGCR inhibitor, when combined with radiotherapy, increased persistent DNA double-strand breaks (DSBs), induced senescence, and enhanced the acute effects of radiation on radioresistant melanoma tumors 165. Seshacharyulu P et al. reported that farnesyl pyrophosphate synthase (FDPS), a branching enzyme of the cholesterol synthesis pathway, was overexpressed in pancreatic ductal adenocarcinoma tissues and was associated with poor radiotherapy response and survival 164, 166. Inhibition of FDPS by zoledronic acid (Zol) exerted radiosensitization by affecting Rac1 and Rho prenylation, thereby modulating DNA damage and radiation response along with improved systemic immune cell activation 166.

### Lipid droplet metabolism

Lipid droplets (LDs) are one of the major intracellular sites for the storage of triglycerides and cholesterol. When glucose is insufficient, cells use lipid hydrolases or the autophagy pathway to breakdown lipids in LDs and then transport FAs into mitochondria to provide energy through fatty acid oxidation 167. Compared with normal cells, tumor cells continuously synthesize and take up large amounts of lipids, and store excess lipids in LDs to provide energy in a "starved" state 167-169. According to some reports, large numbers of gene mutations and the unique microenvironment of tumors endow many metabolic enzymes (e g. phosphoenolpyruvate carboxykinase 1, and choline kinase α2) with protein kinase activity, and promote the formation and degradation of LDs. Additionally, the metabolic processes of LDs are also associated with radiosensitivity 167, 168. It has been reported that inhibiting diacylglycerol acyltransferase 2 (DGAT2), an enzyme in the LD biogenesis process, by PF-06424439 reduced LD content, inhibited cell migration, and sensitized MCF7 breast cancer cells to radiation 170. In addition, LD content is closely related to iron metabolism, especially ferritin heavy chain (FTH1). In breast and lung cancer cells, silencing the FTH1 gene downregulated the amounts of LDs and induced radioresistance, and FTH1 overexpression as well as iron-chelating treatment by dseferoxamine were able to restore these effects 171.

## Nucleotide metabolism and cancer radioresistance

To satisfy the demands of rapid proliferation, tumor cells require large amounts of nucleotides to produce DNA and RNA. Nucleotides consist of purines and pyrimidines, both of which can be synthesized through two pathways: de novo synthesis and the salvage pathway. Although salvage pathways can recycle free purines and pyrimidines for nucleotide biosynthesis, most proliferating cells, especially cancer cells, still synthesize nucleotides via the de novo pathway. Substrates for de novo nucleotide synthesis come from ribose sugars produced by the PPP as well as amino acids and their derivatives, in which one-carbon metabolism is critical. In the de novo synthesis of purines, carbon and nitrogen atoms from glycine, glutamine, aspartic acid, and 10-CHO-THF are sequentially added to ribose 5-phosphate (R-5-P), of which phosphoribosyl pyrophosphate amidotransferase (PPAT), inosine monophosphate dehydrogenase (IMPDH), and ribonucleotide reductase (RNR) are the key rate-limiting enzymes 9. The de novo pyrimidine pathway utilizes substrates such as glutamine, carbon dioxide, and aspartic acid to synthesize orotate, and then reacts with PPRP to generate UDP, which is further converted to CDP and dTMP by RNR and thymidylate synthase (TS) 9. At present, most of the metabolism-related anticancer drugs in the clinic mainly focus on nucleotide metabolism, especially to interrupt DNA synthesis 172. For example, methotrexate, a dihydrofolate reductase (DHFR) inhibitor, is widely used in tumor chemotherapy; and pemetrexed is used for the treatment of non-small cell lung cancer targeting TS and AICAR convertase.

Since radioresistant cancer cells require robust DNA repair capacity to compensate for radiation-induced DNA damage, enhanced nucleotide metabolism is needed in these cells. It has been reported that in glioma, efficient de novo nucleotide synthesis promotes the maintenance of tumor-initiating cells, which contributes to treatment resistance and tumor recurrence 173, 174. Fu S et al. found that glutamine synthetase (GS), regulated by STAT5, was specifically overexpressed in radiation-resistant nasopharyngeal carcinoma and glioma cells, negatively correlating with treatment outcome 64. The activation of GS increased the level of glutamine, which enhanced the metabolic flux of nucleotides and accelerated DNA repair via homologous recombination (HR), finally leading to radioresistance 64. In addition, MUC1, an oncogene overexpressed in multiple solid tumors, was reported to reconnect the metabolic network to the pentose phosphate pathway, mediating the activation of nucleotide production and contributing to radiation resistance in pancreatic cancer cells 33. After radiotherapy, these MUC1-expressing cells treated with 3-bromopyruvate, a glycolysis inhibitor, can abrogate the levels of metabolites in glycolysis, PPP and nucleotide biosynthesis pathways, resulting in radiosensitization by preventing DNA damage repair 33.

### Purine metabolism

By analyzing the metabolomic information of GBM, researchers have discovered that purine metabolites, especially guanylate, are strongly associated with radioresistance 175. Further study showed that purine metabolites induced radioresistance mainly by promoting the repair of radio-induced DNA double-stranded breaks (DSBs) 175. Inhibiting GTP synthesis with MMF, an IMPDH1 inhibitor, could sensitize radioresistant GBM cells in vitro and in vivo 175, 176. Moreover, it is reported that tanshinone I, a PPAT inhibitor, significantly improved the radiation sensitivity of radioresistant lung cancer cells 177. These findings indicate that inhibiting de novo purine synthesis may be a promising strategy to overcome therapy resistance.

### Pyrimidine metabolism

In glioblastoma, upregulation of proliferating cell nuclear antigen (PCNA)-associated factor (PAF) promoted the biosynthesis of pyrimidine metabolites, especially orotate, which maintained glioma stem cells self-renewal and is associated with radioresistance 178. 5-Fluorouracil (5-FU), an analog of pyrimidine nucleoside, could enhance the radiosensitivity of colorectal cancer cells by inhibiting the activity of TS 179, 180. And researchers developed multiple nanoparticles as carrier of 5-FU, which could enhance radio-sensitivity in cancer cells 181, 182. Turnbull T et al. found that nanoparticle-induced impairment enhanced DNA damage by down-regulating repair proteins such as TS 183. In addition, osalmid, a novel identified RNR inhibitor, have been proved to enhance radiosensitivity of esophageal cancer 184.

## Others

In addition to glucose, lipid, amino acid and nucleotide metabolisms reprogramming, the metabolism of metal ions such as copper, iron and calcium are also involved in the regulation of malignant tumor progression and treatment resistance. Ferroptosis, a recently discovered form of cell death driven by iron-dependent lipid peroxidation, plays an important role in tumor radiotherapy. Recent evidence suggests that the synergistic effect of radiotherapy and immunotherapy is associated with increased susceptibility to ferroptosis. Combined radiotherapy and immunotherapy down-regulate SLC7A11, which is mediated by the DNA damage-activated kinases ATM and IFN-γ, resulting in decreased cystine uptake, increased ferroptosis, and limited tumor growth 182. Cancer cells upregulate the expression of SLC7A11 and GPX4 to protect cells from radiation-induced ferroptosis, contributing to radioresistance 185.

Recently, the team of Todd R. Golub from the Broad Institute of Harvard and MIT discovered a new type of cell death, copper death, which has attracted great attention 186. Further research revealed that FDX1 is a key regulator of copper death and an upstream regulator of protein fatty acylation 186. Due to the abundance of FDX1 and fatty acylated proteins, which are highly correlated with a variety of human tumors, copper ionophores may be potential therapeutics against cancer cells with such metabolic signatures. Furthermore, Yang M et al. found that intracellular copper accumulation could promote radiotherapy resistance in multiple cancers including hepatic carcinoma, nasopharyngeal carcinoma and colorectal cancer 187. Mechanistically, the accumulation of intracellular copper ions induced by the downregulation of COMMD10 can inhibit the ubiquitin-mediated degradation of HIF-1α, subsequently increasing the transcription and translation of ceruloplasmin, which forms a positive feedback regulation loop of HIF-1α/CP to inhibit ferroptosis, thereby promoting the radiotherapy resistance of liver cancer cells 187.

## Discussion

It is worth noting that the development of radioresistance is not only associated with tumor cells, but also involves various cells in the tumor microenvironment (e g, immune cells, fibroblasts, endothelial cells, etc.). Rao E et al. reported that all-trans retinoic acid can induce inflammatory macrophages, which induce effector T-cell infiltration and enhance the effector T-cell to regulatory T-cell ratio, overcoming the radioresistance of solid tumors 188. Compared with radiosensitive nasopharyngeal carcinoma tissues, radioresistant cancer cells had more infiltration of cancer-associated fibroblasts, which reduced radiation-induced DNA damage through the IL-8/NF-κB pathway and promoted tumor cells to acquire radiation resistance 189. The protein kinase CK2 is involved in IR-induced cytokine production by endothelial cells, which ultimately leads to radiation resistance in non-small cell lung cancer cells 190. Moreover, Yang X et al. found that radiation-induced lactate enhanced the immunosuppressive phenotype of pancreatic cancer myeloid-derived suppressor cells (MDSCs), and lactate blockade restored antitumor T-cell responses and effectively inhibited tumor progression after radiotherapy 46. MDSCs with high expression of arginase-1 have been reported to deplete arginine to inhibit NO synthesis in macrophages, developing radioresistance 107. These results suggest a significant connection between metabolic reprogramming and radioresistance in the TME. However, the specific molecular mechanism of tumor cell radioresistance of metabolites (e g. lactate, inflammatory factors, etc.) produced by cells in the TME remains unclear. Therefore, future studies on radioresistance can focus on the metabolic link between the tumor and TME.

With a deeper understanding of the complexity of tumor metabolism, it is not difficult to realize that different metabolic pathways weave into a large metabolic network, in which they are interconnected and regulated by each other, synergistically promoting cancer progression. For instance, Yuan Fang et.al. demonstrated that radioresistant liver cancer cells are highly addictive to glucose 191. However, these glucose molecules did not enter the conventional glycolytic pathway but promoted the synthesis of cardiolipin, which inhibited the release of radiation-induced cytochrome C, blocked the initiation of apoptosis, and ultimately enhanced radiation resistance. This study reveals a new mechanism by which glucose links cardiolipin anabolism to mediate radiation resistance in liver cancer.

Flexible changes in cellular metabolism satisfy the demands of tumor tissue growth, and we have found that there is a large amount of metabolic heterogeneity in tumors. The metabolic heterogeneity of tumors can be mainly manifested in three aspects: one is between tumor cells and nontumor cells; the second is between different types of tumors; and the third is during the development process of tumor resistance. During tumor invasion, cancer cells activate glycolysis to release lactic acid and other organic acids to acidify the extracellular environment and promote the degradation of ECM, which is beneficial to tumor cell invasion 192. However, when they become circulating tumor cells, the metabolic phenotype switches to PPP, and produces sufficient NADPH and GSH to counteract ROS 192. Furthermore, it is interesting to note that different cell groups have different nutrient uptake propensities from the TME, with glutamine and fatty acids mainly distributed to cancer cells, while glucose is preferentially provided to immune cells 66. Therefore, how tumors balance the metabolism of glucose and glutamine is a key issue. These studies have shown that there is metabolic heterogeneity in the radioresistance of tumor cells, and targeting only a single metabolic target is not enough to overcome this radioresistance. In the future, we hope to find common metabolic targets in different radiation-resistant tumors and implement pancancer therapy based on these findings.

In general, current studies on sensitizing cancer cells to radiation based on metabolic reprogramming are mainly focused on metabolic enzyme inhibitors and nutrient deprivation. Considering the complexity and heterogeneity of metabolism, future studies are needed to deeply explore the relationship between the mechanism of radioresistance and metabolism and distinguish tumor subtypes with unique metabolism alterations. To identify and sensitize radioresistant cancers early, it is necessary to explore new tracers and sensitizers targeting metabolism in preclinical and clinical studies.

## Funding

This work was supported by Natural Science Foundation of China (NSFC 12275178, 82000939, 82203260), The Science and Technology Commission of Shanghai (20DZ2270800, 22Y31900700), Shanghai Pujiang Program22PJD036.

## Figures and Tables

**Figure 2:**
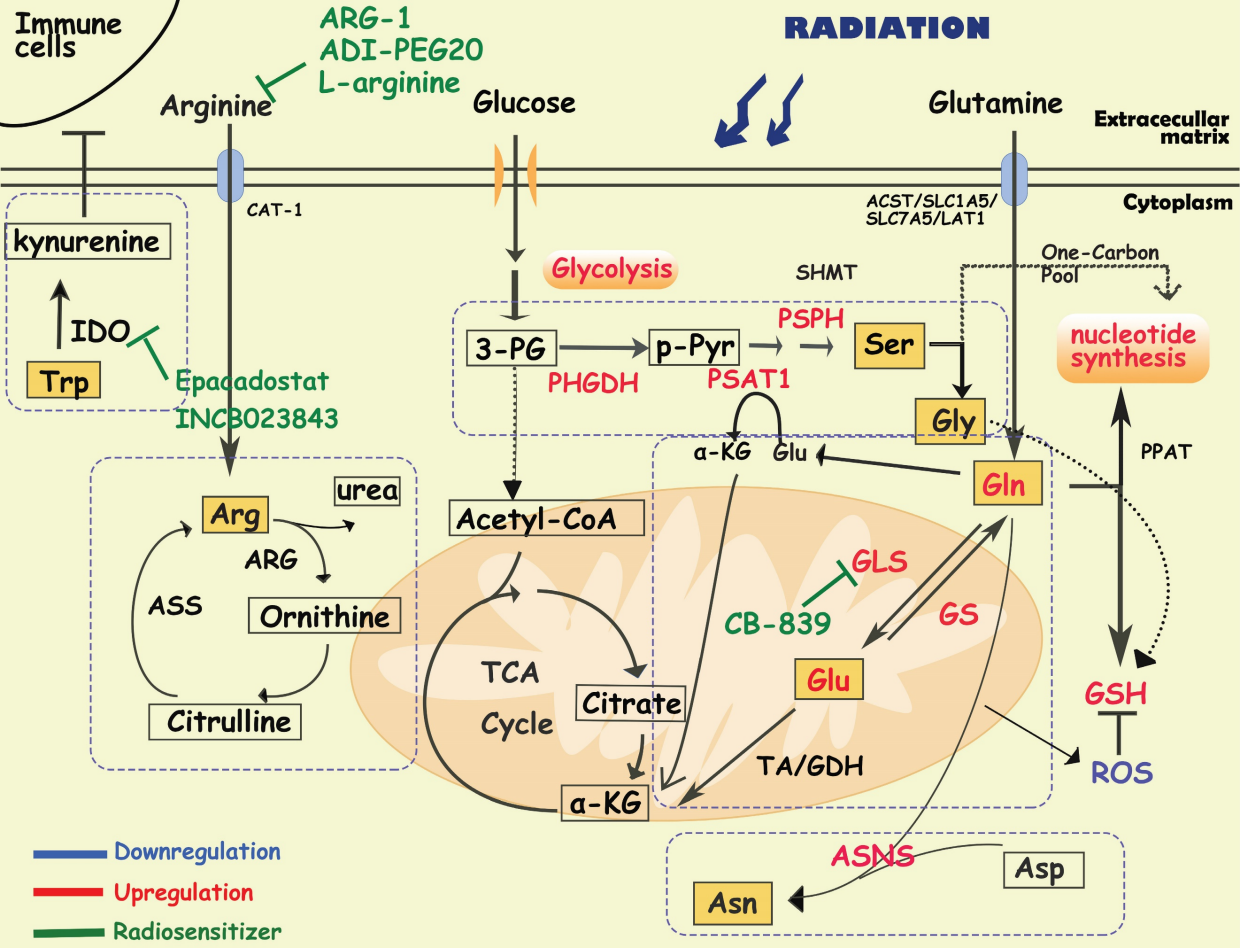
** Diagram of amino acids metabolism involved in cancer radioresistance and its radiosensitizers.** In response to radiotherapy, tumor cells enhance the metabolism of amino acids such as glutamate, serine, and asparagine, which provides the biomacromolecules and other materials required for nucleotides, GSH and energy production, prolonging the cancer cell survival. Tumor cells regulate glutamine metabolism by self-balancing the activities of GLS and GS, promoting cell resistance to radiation. Abnormally activated glycolysis also indirectly allows tumor cells to enhance serine synthesis, increasing one-carbon metabolic flux. In addition, amino acids, such as arginine, asparagine and tryptophan, also play important roles in the formatino of cancer radioresistance, which have been proved to be the effective targets for radiosensitization.

**Figure 3:**
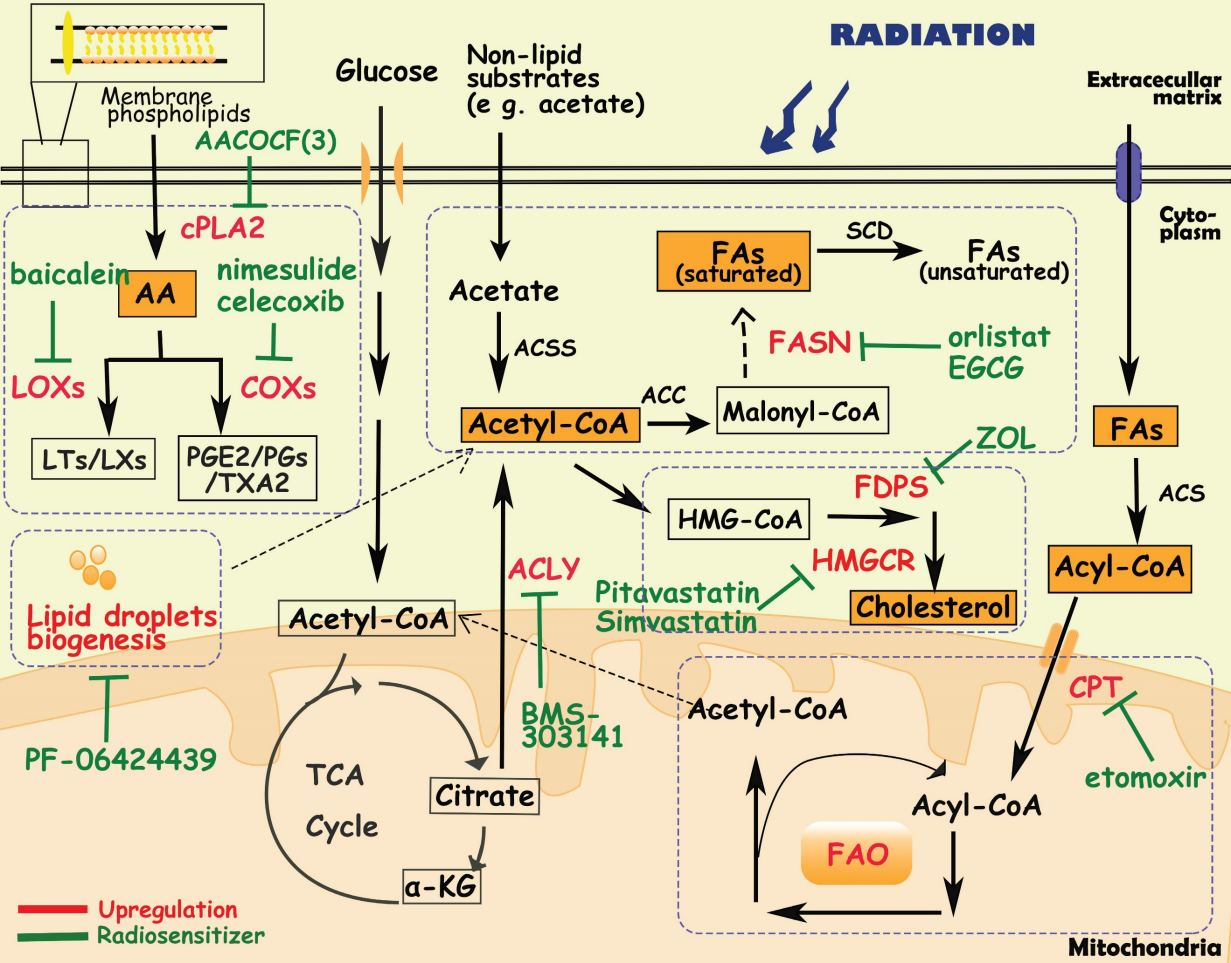
** Diagram of lipids metabolism involved in cancer radioresistance and its radiosensitizers.** Cancer cells develop radioresisitance via reprogramming lipids metabolism including fatty acids, cholesterol, arachidonic acids and lipid droplets. In lipids metabolism, acetyl-CoA is an important bridge connecting different metabolisms. In fatty acids metabolism, tumor cells upregulate the expression of FASN/CPT/ACLY to facilitate the synthesis and oxidation of FAs. In arachidonic acids metabolism, inhibition of metabolic enzymes (e g. cPLA2, LOXs, COXs) is proven to sensitize tumor cells to radiotherapy. Additionally, lipid droplets and cholesterol metabolism are positively correlated with radioresistance as well.

**Figure 4:**
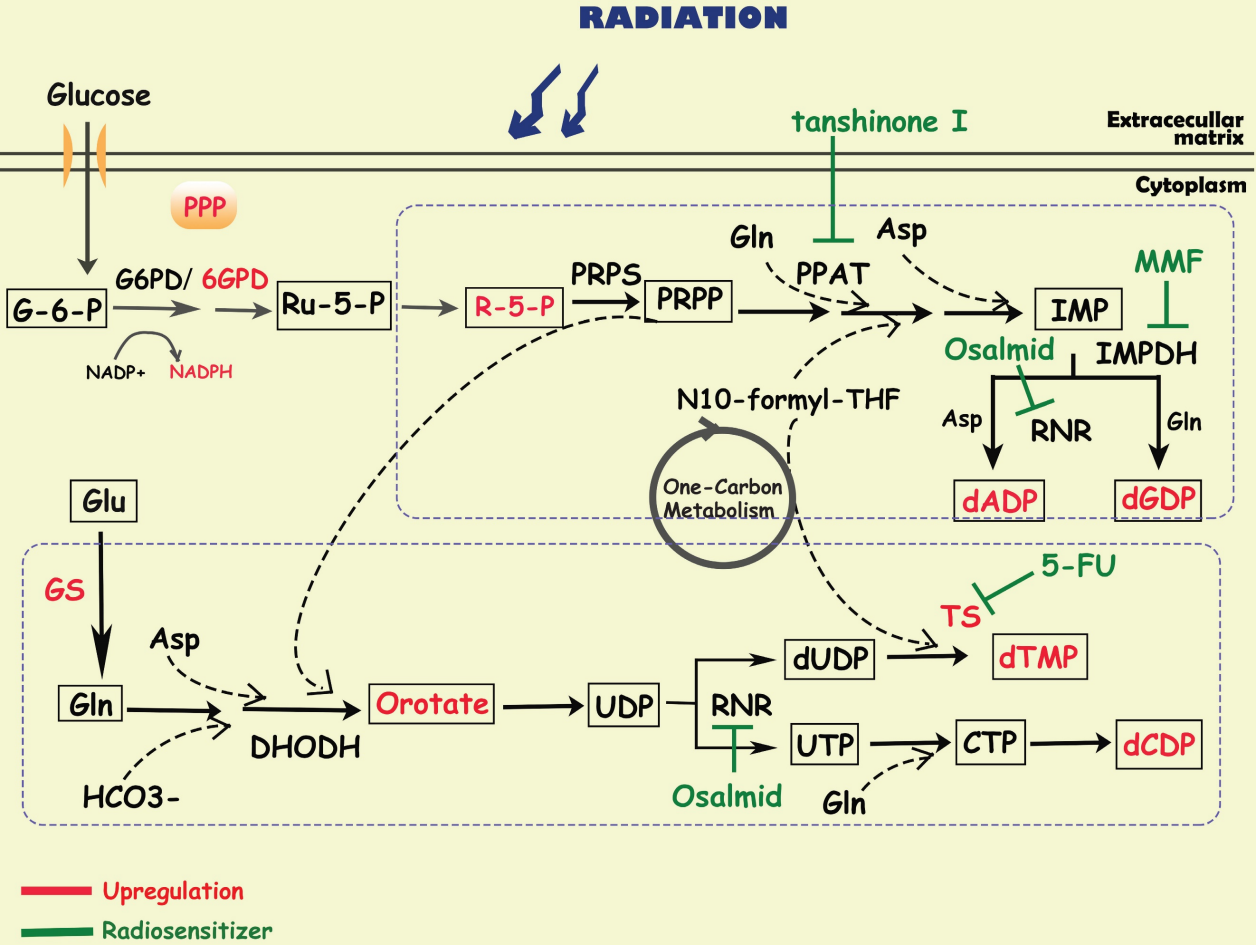
** Diagram of nucleotide metabolism involved in cancer radioresistance and its radiosensitizers.** DNA damage repair is one of the major mechanisms by which tumor cells develop radioresistance, requiring the accumulation of a large amount of nucleotides. To promote tumor cell survival during radiotherapy, activated glucose metabolism (e g. glycolysis, PPP) and amino acid metabolism (e g. glutamate, asparagine, serine) provide sufficient substrates and energy to accelerate purine and pyrimidine synthesis. Simultaneously, related enzymes in the de novo nucleotide synthesis pathway (e g. TS/PPAT/IMPDH) have been shown to be targets for radiosensitization.

**Table 1:** Metabolism associated targets in radioresistance and the radiosensitization methods.

Items	Targets	Radiosensitizer	Cancer cells	Function	Ref.
**Glycose metabolism**	*GLUT1*	WZB117	Breast cancer	-	31
	*HK2*	2-DG	Cervical cancer	Increase ROS level	32
		3-bromopyruvate	Pancreatic cancer	Reduce nucleotide biosynthesis and enhance DNA damage	33
	*PKM2*	TEPP46	Triple negative breast cancers	Deplete breast cancer stem cells	37
	*LDHA*	Oxamate	Non-small cell lung cancer	Induced ROS accumulation and cellular ATP depletion, increase DNA injury	41
	*MCT1*	AZD3965	Small cell lung cancer	-	47
	*MPC1*	7AAC2	Cervix cancer, Pharynx squamous cell carcinoma, Breast cancer	Reduce hypoxia	49
	*PDK*	Dichloroacetate (DCA)	Triple negative breast cancers	Increase ROS production	54
		Diisopropylamine dichloroacetate (DADA)	Esophageal squamous cell carcinoma	Increase intracellular levels of ROS	56

**Amino acid metabolism**	*GLS*	Terlaglenastat (CB-839)	Cervical cancers	Decreased total glutathione	70
			Head and neck squamous cell carcinoma	Increased oxidative stress and DNA damage	71
			Lung cancer	Reduce GSH	72, 75
	*Arginine*	ARG-1	Glioblastoma	-	109
			Epithelial cancer	-	110
		ADI-PEG20	Pancreatic cancer	Promote apoptosis	111
		L-arginine	Brain metastases from solid tumors	Reduce glycolysis and impact ATP and NAD levels	112
	*IDO-1*	Epacadostat	Colorectal cancer	Reduce production of kynurenine; potentiated Th1 cytokines and myeloid cell-modulating factors in TME	118
		INCB023843	Lung cancer	Reducing numbers of IDO1-expressing MDSCs in TME	117

**Lipid metabolism**	*ACLY*	BMS303141	Head and neck squamous cell carcinoma	Inhibit DNA damage repair	127
	*FASN*	Orlistat	prostate cancer	Decrease NF-κB activity	135
		Epigallocatechin gallate (EGCG)	Nasopharyngeal carcinoma	-	137
	*CPT-1*	Etomoxir	Glioblastoma multiforme	Boost macrophage phagocytosis	143
			Breast cancer	-	148
			Nasopharyngeal carcinoma	-	146
	*cPLA2*	AACOCF(3)	Ovarian cancer	Inhibit activation of pro-survival Akt signaling and enhance cell death	156
	*12-LOX*	Baicalein	Prostate cancer	-	158
	*COX-2*	Nimesulide	Lung cancer	Increased apoptosis	193
		Celecoxib	Lung cancer	Regulate radiation-induced G2-M arrest	194
			Esophageal squamous cell carcinoma	-	161
		Celecoxib- Afatinib combination	Non-small cell lung cancer	Regulate cell sensitivity to apoptosis	195
	*HMG-CoA*	Pitavastatin (Livalo)	Melanoma tumors	Delay DNA repair, enhance acute effects of radiation	165
		Simvastatin	Prostate cancer	Compromise DNA double-strand break repair	196
	*FDPS*	Zoledronic acid (Zol)	Pancreatic ductal adenocarcinoma	Attenuate DNA damage repair and immunosuppressive signaling	166
	*DGAT2*	PF-06424439	Breast cancer	Reduce LD content	170

**Nucleotide metabolism**	*IMPDH1*	MMF	glioblastoma	Inhibit GTP synthesis	175
	*PPAT*	Tanshinone I	lung cancer	Well-docked into the active pocket of the structure of PPAT	177
	*TS*	5-FU	Colorectal cancer	-	180
	*RNR*	Osalmid	Esophageal cancer	Enhance DNA damage, apoptosis, and senescence	184
